# Active pulmonary tuberculosis presenting with acute respiratory failure

**DOI:** 10.1002/rcr2.460

**Published:** 2019-07-16

**Authors:** Satish Chandra Kilaru, Sudhir Prasad, Hemanth Kilaru, Raghavender Reddy Anneela, Ashfaq Hasan, Eshwar Chandra Nandury

**Affiliations:** ^1^ Department of Respiratory Medicine Prathima Institute of Medical Sciences Karimnagar India; ^2^ Internal Medicine, Pulmonology and Critical Care Medicine Global Hospitals Hyderabad India; ^3^ Respiratory Medicine Deccan College of Medical Sciences Hyderabad India; ^4^ Department of Radiology Virinchi Hospitals Hyderabad India

**Keywords:** Active tuberculosis, bronchogenic pulmonary tuberculosis, India, respiratory failure

## Abstract

Four patients with active pulmonary tuberculosis (PTB) presenting with respiratory failure are reported here. Bronchogenic PTB, simulating an acute febrile illness or diffuse interstitial lung disease with short duration of symptoms, as a cause of acute respiratory failure is less recognized. If diagnosed and treated early, it has good prognosis. Three of the four patients presented here had an acute presentation with fever, dyspnoea, and hypoxemia with diffuse infiltrative lesions on radiography, and the other younger patient presented predominantly with lobar consolidation. These patients presenting with respiratory failure required intensive care management, and a diagnosis was made with bronchoalveolar lavage fluid and transbronchial lung biopsy. All four patients promptly received antitubercular therapy, showed clinicoradiological improvement, and were stable at 1 year follow up.

## Introduction

Tuberculosis continues to be a major public health problem causing ill health to approximately 10 million people each year. It is 1 of the 10 causes of death worldwide from a single infectious agent, causing an estimated 1.3 million deaths among human immunodeficiency virus (HIV)‐negative people in 2017. India, according to the World Health Organization (WHO), is considered one of the high‐burden countries for tuberculosis (TB), with an estimated total TB incidence of 204/100,000 and mortality of 31 per 100,000 population 1.

Among notified cases of TB in India, only 60% of the pulmonary TB cases were bacteriologically confirmed, and there was an overall reduction of mortality with TB treatment during 2000–2017 of 42%, emphasizing the need for early diagnosis and treatment with antitubercular therapy (ATT) 1.

Acute respiratory distress syndrome (ARDS) is known to be one of the complications of miliary TB and also in bronchogenic PTB. Bronchogenic PTB is a less‐recognized cause of acute respiratory failure, simulating an acute febrile illness or diffuse interstitial lung disease, with a short duration of symptoms 2. The incidence of this clinical situation was reported to be 1.5–1.9% in earlier studies 3, 4. Mortality was reported to be 75% in patients with symptoms for more than 2 weeks 5.

Here, we present case reports of four patients of bronchogenic PTB with acute respiratory failure who were treated successfully.

## Case Series

### Case 1

**Figure 1:**
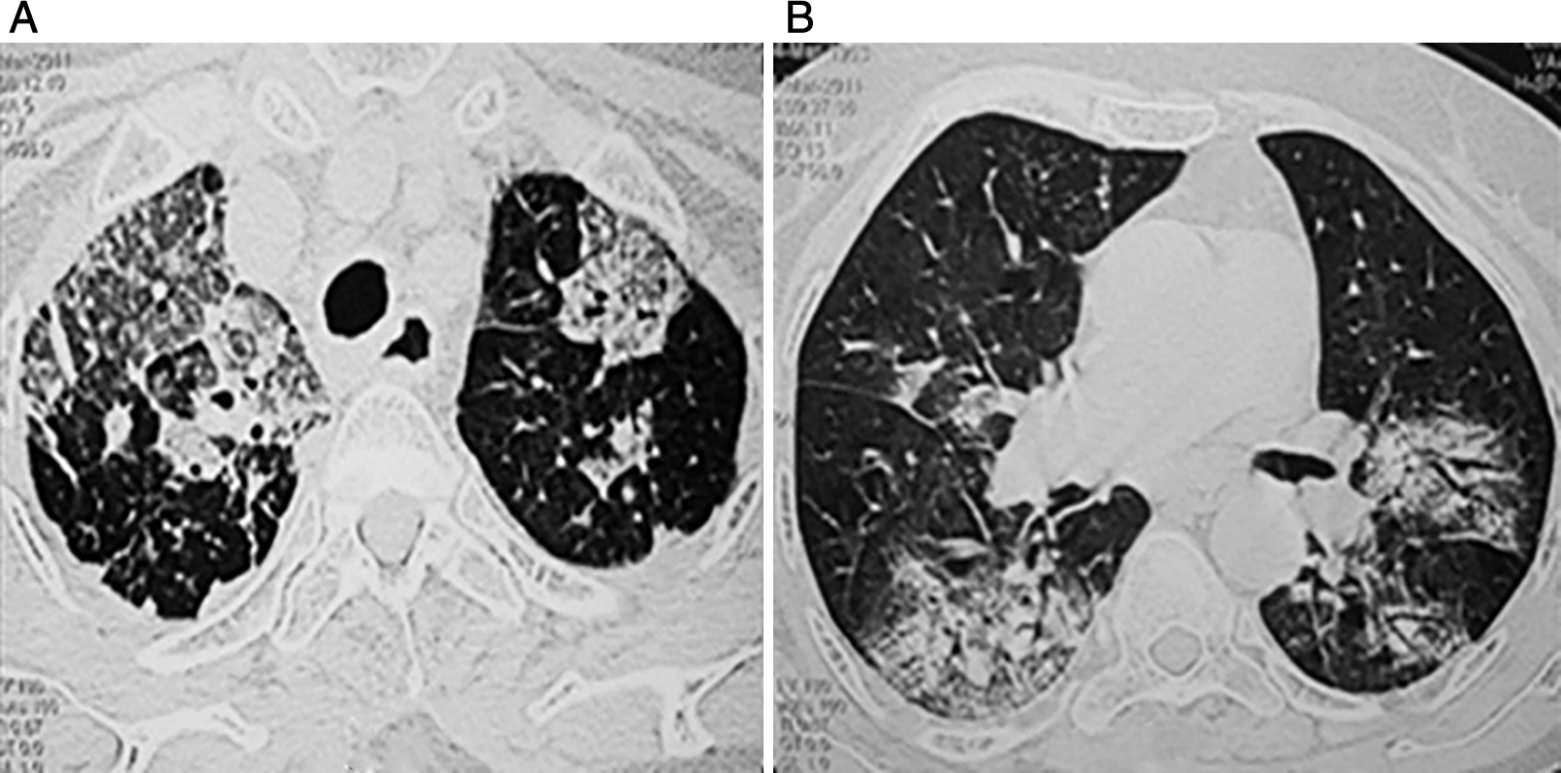
(A, B) High resolution computed tomography (HRCT) sections obtained through the apical and basal regions of both lungs demonstrate large ground‐glass opacities with septal thickening, tiny nodules, and a few larger peribronchovascular nodules.

A 63‐year‐old housewife was admitted to the hospital with cough, fever, and dyspnoea of a duration of more than 2 weeks. She was treated with methylprednisolone and antibiotics for cryptogenic organizing pneumonia (COP) and superadded infection, respectively, in an intensive care unit (ICU) elsewhere, prior to the present admission, along with supplemental O_2_. Her relevant lab data were as follows: SpO_2_: 88% (PaO_2_ 58.1 mmHg), hyponatraemia of 128 mg%, and serum albumin: 2.6 g. High resolution computed tomography (HRCT)‐Chest at previous admission was as shown in Figure 1A, B.

A bronchoalveolar lavage fluid (BALF) smear for acid‐fast bacilli (AFB) was positive, and histopathological examination of transbronchial lung biopsy (TBLB) showed a granulomatous lesion with necrosis consistent with TB. She showed gradual clinicoradiological improvement with ATT and was maintaining normal SpO_2_ at room air more than 12 weeks after her discharge.

### Case 2

**Figure 2:**
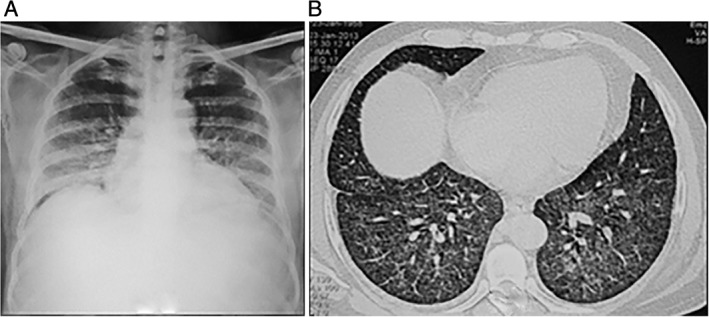
(A) Frontal chest radiograph (PA projection) demonstrates haziness in bilateral mid and lower lung zones with ill‐defined tiny nodular opacities. (B) High resolution computed tomography (HRCT) section of the lungs in the lower lobe region demonstrates multiple tiny perilymphatic and randomly distributed nodules.

A 55‐year‐old male pharmacist, with no previous health issues, was admitted to hospital with fever of a duration of 2 weeks and dyspnoea for a week, with an SpO_2_ of 89% on room air (PaO_2_. 57.9 mmHg), aspartate aminotransferase (AST) of 65 μ/L, total bilirubin of 1.6 mg/dL, platelet count of 89,000, and serum creatinine of 2.1 mg%; radiographic and computed tomography (CT) findings were as shown in Figure 2A, B. In view of the initial clinicoradiological presentation, subacute hypersensitivity pneumonitis was also considered a differential diagnosis. TBLB showed granulomatous lesions with caseating necrosis, consistent with TB. The patient showed rapid clinicoradiological recovery with ATT.

### Case 3

**Figure 3:**
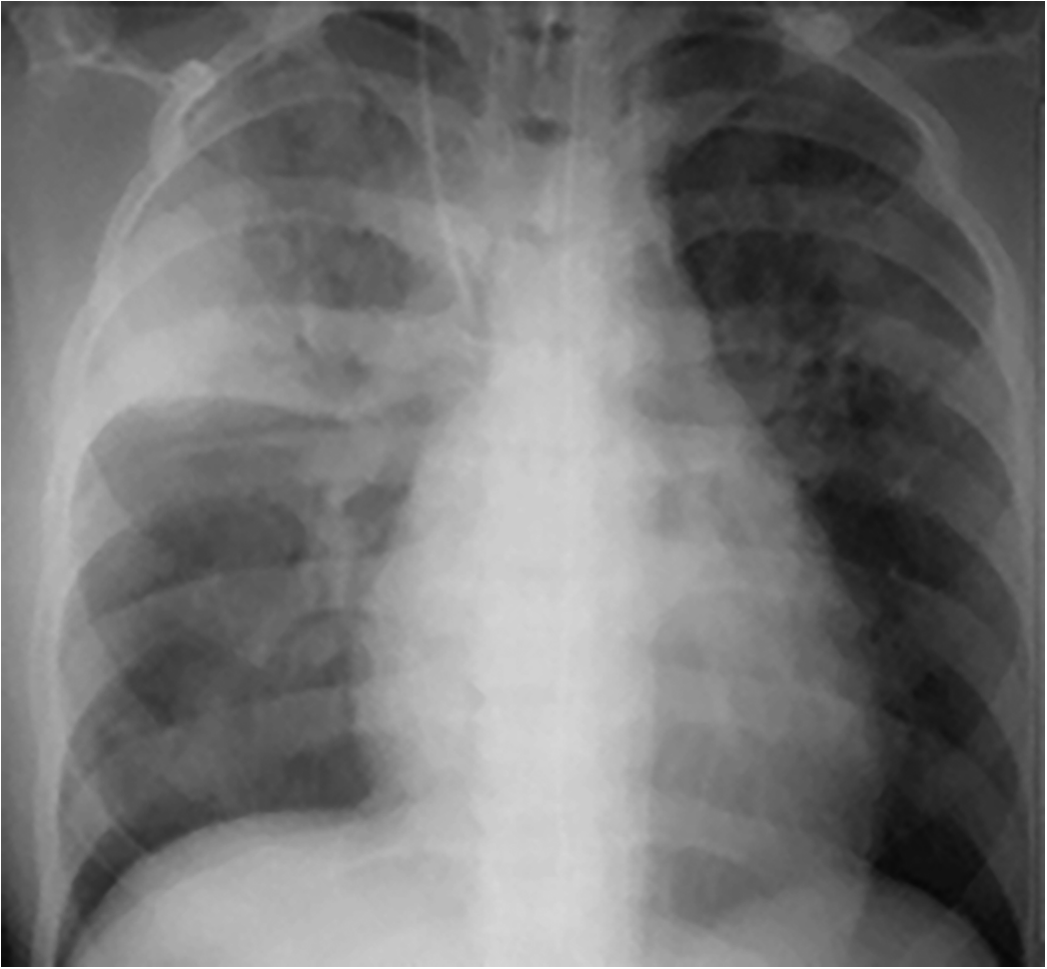
Frontal chest radiograph shows a large area of right upper lobe consolidation with air bronchograms, which is limited inferiorly by the minor fissure. Patchy consolidation is seen in the right lower lung zone and in the left upper and mid‐lung zones in the perihilar region.

An 18‐year‐old female college student presented to the emergency room (ER) with a history of fever, cough for 2 weeks, and rapidly progressing dyspnoea for 2 days. She had a history of contact with TB through her father. She was tachypneic and tachycardic (HR:165 bpm). SpO_2_ was 86% on high‐flow O_2_ with a PaO_2_ of 51.5 mmHg. While being stabilized on non‐invasive ventilation (NIV) in the ER, the patient became severely breathless and had to be intubated and ventilated. Chest X‐Ray showed consolidation involving the right upper lobe, with patchy consolidation in the left upper lobe (Fig. 3). Endotracheal tube (ET) secretions returned positive for AFB. ATT was started along with supportive treatment. The patient was extubated successfully on day 4 and made an uneventful recovery.

### Case 4

**Figure 4:**
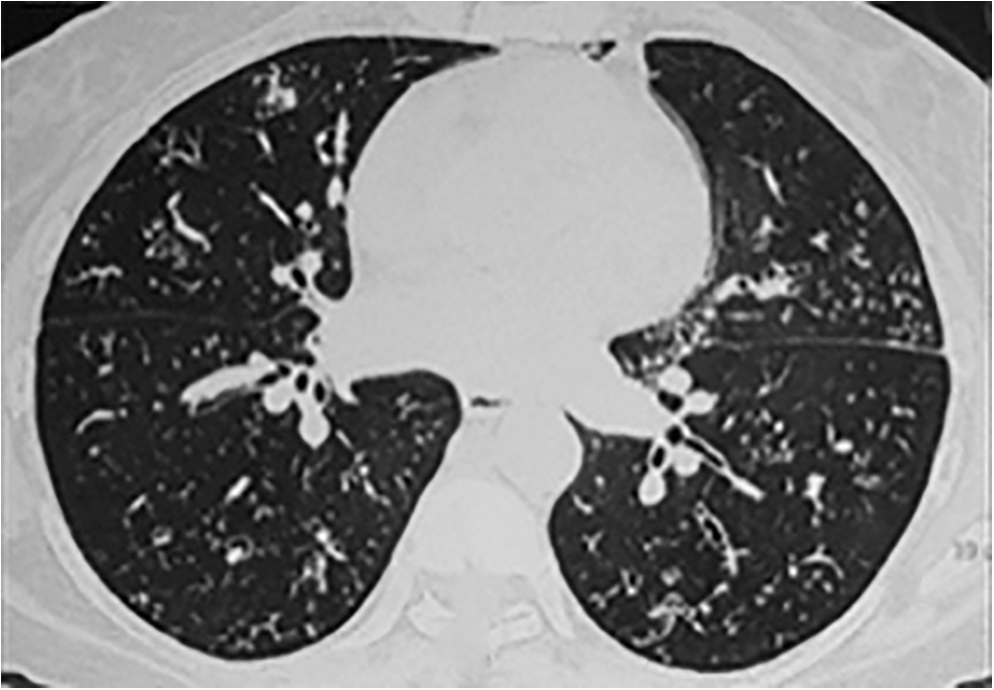
High resolution computed tomography (HRCT) section through the lower lobes demonstrates multiple tiny centrilobular nodules with a tree–in‐bud appearance and mild cylindrical bronchiectasis bilaterally.

A 49‐year‐old female with a history of asthma and allergic bronchopulmonary aspergillosis (ABPA), being treated with long‐term inhaled corticosteroids (ICS) + long‐acting beta‐agonist (LABA) and alternate‐day prednisolone, was admitted with cough, low‐grade fever for a month, and increasing dyspnoea for 2 weeks. SpO_2_ at room air was 88% (PaO_2_ of 58 mmHg). Her HRCT‐Chest findings were as shown in Fig. 4. She was started on ATT after her BALF tested positive for AFB. In addition, her TBLB showed granulomatous lesions with caseating necrosis, and her BALF culture was later found to be positive for *Mycobacterium tuberculosis* complex. She showed gradual clinicoradiological improvement, and her PaO_2_ returned to normal with SpO_2_ (at room air) of 96% at 6‐month follow up.

## Discussion

Three of the four patients presented here had acute presentation with fever, dyspnoea, and hypoxemia with diffuse infiltrative lesions on radiography. The other younger patient (Case 3) presented with lobar consolidation, simulating non‐tuberculous pneumonia with acute respiratory failure.

The clinicoradiographic profiles of these patients suggest bronchogenic PTB predisposing to acute respiratory failure. Similar clinical presentations were reported in 5 of the 17 patients in a study by Choi et al. 2.

Presentation as an acute infective episode or interstitial pneumonia simulating early ARDS or acute respiratory failure, such as in our Case 1, should prompt clinicians to consider PTB. Treatment with steroids, prior to present admission, might have contributed to initial clinicoradiological improvement in this patient, but a gradual recovery with ATT was noted over 4 weeks. Corticosteroids in the treatment of PTB with bronchogenic dissemination and respiratory failure may be beneficial as a non‐specific anti‐inflammatory therapy 6.

Presentation as acute febrile illness, progressing to dyspnoea of less than 2 weeks, could be a reflection of hypoxemic respiratory failure, such as in Case 2. Chest X‐ray showing infiltrates and associated hypoxemia should not deter the clinician from considering PTB. This poses a diagnostic dilemma during seasonal “flare‐ups” of viral respiratory infections. Our initial suspicion was of a viral interstitial pneumonia with acute respiratory failure and was managed in the ICU. We relied on the combination of BALF AFB smear and histopathology of TBLB specimens and, later, AFB culture for diagnosis because the Xpert MTB/RIF assay was unavailable during this period of study. Addition of the Xpert assay would have prevented the need for TBLB in such patients.

Case 3 presented predominantly with lobar consolidation and acute respiratory failure, simulating bacterial pneumonia, and had to be mechanically ventilated.

Case 4 showed recent clinicoradiological deterioration with rest‐hypoxemia, which should alert the clinician to consider the possibility of bronchogenic PTB, especially when the patient is on long‐term oral corticosteroids. Exacerbations may be ascribed to either asthma or ABPA in such patients.

Four independent predictors, viz., symptoms of more than 1 month before initiating treatment, hypoalbuminaemia, multiple organ dysfunction, and higher number of pulmonary lobes involved, are independently associated with a higher 30‐day mortality rate 7. Patients with miliary TB presenting as ARDS had a longer duration of illness prior to diagnosis. Delay in treatment initiation may increase mortality in patients with active TB and may predispose to ARDS 8. Case 1, who had hyponatraemia, hypoalbuminaemia, and illness of more than 4 weeks with diffuse lung lesions, fulfils these criteria before diagnosis and for the initiation of treatment. Hyponatraemia was reported to have an increased fatality rate 8. It was considered a predictor of increased mortality in the studies by Levy et al. (33%) and Anderson et al. (60 fold) 3, 9. Similarly, Case 2 had thrombocytopenia, acute kidney injury (AKI), elevated liver enzymes, and diffuse lung lesions with symptoms of a duration of 2 weeks.

Although ARDS is reported in active TB and miliary dissemination, many patients with confluent pulmonary infiltrates (non‐miliary‐PTB) or consolidation with atypical clinical features may present with acute respiratory failure. However, acute respiratory failure associated with PTB was reported to have a good prognosis with 67% survival when compared to 46% in patients presenting with ARDS 3. The above illustrative cases indicate that the presentation of PTB with a short history and hypoxemia has good prognosis, provided they are addressed at an early stage. Presence of respiratory failure with an acute presentation, as discussed, is one of the reasons of delay in diagnosing active TB.

Cause of hypoxemia in non‐miliary PTB is a result of direct injury to alveolar epithelial cells from tubercular antigens through liquefied, caseous lesions. These effects may further be accentuated by bronchogenic spread. A small amount of bacillary antigen is enough to evoke an exudative response in the host and is an important determinant of direct injury 10. The key factor in the above process is the activation of alveolar macrophage. Lipoarabinomannan (LAM), a tuberculous cell wall constituent, similar to the lipopolysaccharide in Gram‐negative sepsis, activates macrophages that trigger the production of tumor necrosis factor (TNF)‐alpha, interleukin (IL)‐1beta, and mRNA from mononuclear phagocytes. Similarly, mycobacterial heat shock protiene‐65 kD and *M. tuberculosis* culture filtrate may incite similar effects 11. In addition, *M. tuberculosis* makes endothelial cells more susceptible to the toxic effects of TNF‐alpha and increases ICAM‐1 expression on endothelial cells. Increased expression of this molecule may allow increased binding of neutrophils to the endothelium 12. In later stages, the spread of infection into the blood may diffusely injure the vascular endothelium. This may cause similar effects seen in indirect injury from sepsis leading to ARDS 13. A combination of these processes would ultimately affect the A‐a O_2_ gradient leading to hypoxemia, thus manifesting as respiratory failure.

As bronchogenic PTB with respiratory failure has a good prognosis, early diagnosis and treatment are imperative to prevent not only morbidity and mortality but also disease transmission.

## Disclosure Statement

Appropriate written informed consent was obtained for publication of this case report and accompanying images.
